# HCV knowledge and attitudes among HIV-negative MSM and MSM living with HIV in China: results from a cross-sectional online survey

**DOI:** 10.1186/s12879-023-08586-1

**Published:** 2023-09-13

**Authors:** Wenqian Xu, Peizhen Zhao, Haiyi Li, Cheng Wang

**Affiliations:** 1 Dermatology Hospital of Southern Medical University, Guangzhou, China; 2 Southern Medical University Institute for Global Health, Guangzhou, China; 3 Guangdong Provincial Center for Skin Diseases and STIs Control, Guangzhou, China; 4 School of Public Health, Southern Medical University, Guangzhou, China

**Keywords:** HCV, Knowledge, Attitude, MSM, China

## Abstract

**Background:**

Men who have sex with men (MSM) are at high risk of hepatitis C virus (HCV) infection, especially for those living with human immunodeficiency virus (HIV). Learning about knowledge of and attitudes towards HCV is essential to inform health promotion interventions development. This is one of very limited studies to examine the level of knowledge and attitudes towards HCV and their determinants among HIV-negative MSM and MSM living with HIV in China.

**Methods:**

A cross-sectional survey was conducted across seven provinces in China from December 2021 to January 2022. All the MSM living with HIV were recruited offline, whereas the recruitment ratio for HIV-negative MSM was half online and half offline. Data on socio-demographic characteristics, sexual behaviors, knowledge about HCV, and attitude towards HCV from participants were collected through the online survey. Univariate and multivariable logistic regressions were used to determine the associated factors.

**Results:**

Only 39.3% (33/84) of HIV-negative men and 44.0% (37/84) of men living with HIV had a good level knowledge about HCV. Nearly one-third (32.1%, 27/84) of HIV-negative men and 41.7% (35/84) of men living with HIV reported a positive attitude towards HCV. For HIV-negative men, positive attitude towards HCV was associated with the multiple sexual partners (aOR: 5.8, 95%CI:1.9–18.1) and the use of recreational substances (aOR: 3.1, 95%CI: 1.0-9.4). For men living with HIV, knowledge about HCV was associated with disclosing sexual orientation to healthcare providers, family or friends (aOR: 7.0, 1.9–26.0), the multiple sexual partners (aOR: 0.2, 0.1-1.0), the use of recreational substances (aOR: 3.7, 95%CI: 1.1–13.1) and the HBV testing history (aOR: 7.3, 95%CI: 1.6–32.7); positive attitude towards HCV was associated with the use of recreational substances (aOR: 3.1, 95%CI: 1.1-9.0).

**Conclusions:**

The majority of Chinese MSM showed an inadequate knowledge of and negative attitude towards HCV irrespective of HIV infection status. More tailored education campaigns and multicomponent interventions are required to be targeted on MSM, and more researches are also needed to inform how best to address the negative attitudes of this population towards HCV.

**Supplementary Information:**

The online version contains supplementary material available at 10.1186/s12879-023-08586-1.

## Background

Hepatitis C virus (HCV) infection remains a significant public health concern globally [1]. Untreated HCV infection contributes to a variety of severe complications such as liver cirrhosis and hepatocellular carcinoma [2]. According to the World Health Organization (WHO), at least 75 million people living with HCV, and 70,000 die each year from an HCV-related cause [3]. Men who have sex with men (MSM) are disproportionately affected by HCV infection, especially for MSM living with human immunodeficiency virus (HIV). In 2015, a global systematic review estimated that the HCV seroprevalence among MSM living with HIV was 6.3% (95% CI: 5.3–7.5) compared to 1.5% (95% CI: 1.0-2.1) in HIV-negative MSM worldwide [4]. In China, the estimated prevalence of HCV in general MSM was 0.67%, whereas the prevalence was about eight times higher among Chinese MSM who were HIV-positive (8.4%) [5].

The introduction of high effective direct-acting antiviral (DAA) therapy has significantly improved HCV treatment outcomes and reduced HCV transmission at the population level [6]. WHO recommends therapy for all HCV-infected individuals, regardless of disease stage [7]. However, MSM encounter barriers along the HCV continuum care, from early testing to achieve a sustained virologic response (SVR) [8]. Previous studies found that lack of HCV-related knowledge was the main reason among MSM [9, 10]. Negative attitudes, lack of self-efficacy, and social stigma also hindered the access and uptake of HCV healthcare services among MSM [10–12]. This situation could be worsen due to ongoing COVID-19 restrictions [13]. Numerous studies indicated that improving knowledge was effective in increasing uptake and adherence to antiretroviral therapy [14–18]. However, the studies regarding knowledge of HCV among MSM in China were limited.

This study aimed to explore and examine the knowledge and attitudes about HCV and their determinants among HIV-negative MSM and MSM living with HIV in China to provide effective basis for the development of tailored intervention program.

## Methods

### Study design and participants

This was a cross-sectional study of baseline data from two parallel randomized controlled trials (RCT), which aimed to evaluate the effectiveness of providing HCV self-testing to increase testing uptake among Chinese MSM: one among HIV-negative MSM and another among MSM living with HIV. Detailed information can be found in the protocol (Appendix p2-23. Study protocol). The baseline survey, was conducted online from December 2021 to January 2022 across seven provinces (Shandong, Guangdong, Liaoning, Qinghai, Hubei, Chongqing and Hebei) in China. Prospective participants were recruited in cooperation with seven local community-based organizations (CBOs). All the MSM living with HIV were recruited offline, whereas the recruitment ratio for HIV-negative MSM was half online and half offline. The offline recruitment was implemented at the MSM-led clinic sites. Staffs in the clinic sites provided information on the study to men who were seeking routine testing and care services, and enrolled HIV-negative men based on their test reports in the past three months and men living with HIV according to their HIV-positive test reports, and then send them links to access the online survey. For the online recruitment, study messages and a survey link were promoted through the chatting platforms or chat groups of the social media software by the CBOs staffs, and participants were required to upload a test result in the past three months to validate their HIV-negative status during the eligibility screening procedure. The survey was administered through Wenjuanxing (Changsha Haoxing Information Technology, China), a professional online questionnaire platform that can provide anonymous surveys.

All potential participants who clicked on the survey link were screened for eligibility after signing an electronic informed consent. Inclusion criteria included: born biologically as a male, aged 18 or over, engaged in anal sex with a man, had not been tested for HCV in the past year, and had at least one of the following risk factors in the past year (condomless anal sex or sexually transmitted infection (STI) diagnosed or injection drug use). Eligible participants must provide a working unique mobile phone number and WeChat account to be enrolled. All men who complete the survey received $3 as compensation for their time.

### Measures

#### Socio-demographics characteristics

Sociodemographic information included: age, region of residence, marital status, educational attainment, employment status, annual income, sexual orientation, and sexual orientation disclosure to healthcare providers, family or friends.

#### Attitude towards HCV

Attitude towards HCV was measured by 13 items. For example, one item was “I would not want my child to attend school where one of the students had Hepatitis C.” Each item was 1 if participants agreed and 0 if they disagreed or neutral. The total score ranged from 0 to 13. A higher score indicated a more positive attitude towards people living with HCV. We categorized individuals’ attitudes into negative, neutral, and positive if they received scores of 0–3, 4–8, and 9–13, respectively [19].

#### Knowledge of HCV

Knowledge of HCV infection was measured by 14 items, which was adapted from the HCV knowledge scale developed and validated by Balfour et al. (2009) [20]. Item content areas included: knowledge about HCV transmission, prevention and treatments. Each of the 14 knowledge items was coded 0 for an incorrect answer and 1 for a correct answer, and the total score ranged from 0 to 14. A higher score indicated a better knowledge of HCV infection. We categorized individuals’ knowledge into poor, moderate and good if they received scores of 0–4, 5–9, and 10–14, respectively [19].

#### Sexual behaviors and testing history

Sexual behavioral variables included number of male sexual partners in the past 3 months, condomless sex with male partners in the last 6 months, group sex, and recreational substances use. Testing history included the following: HIV testing including either facility-based testing or self-testing, testing history for syphilis, Hepatitis B virus (HBV), chlamydia and gonorrhea.

### Statistical analysis

Descriptive statistics were used to describe the distribution of the sample regarding socio-demographics, sexual behaviors, HCV-related knowledge, attitudes toward HCV. Chi-square tests were performed to compare differences in knowledge of and attitudes toward HCV across subgroups of respondents by socio-demographic and behavioral characteristics. Univariate and multivariable logistic regressions were conducted to explore the factors associated with knowledge and attitude about HCV. In the multivariable models, we adjusted for age, region of residence, education, marital status, and income. Statistical significance was defined as p < 0.05. All analyses were conducted using R (version 4.1.0).

## Results

**Fig. 1:**
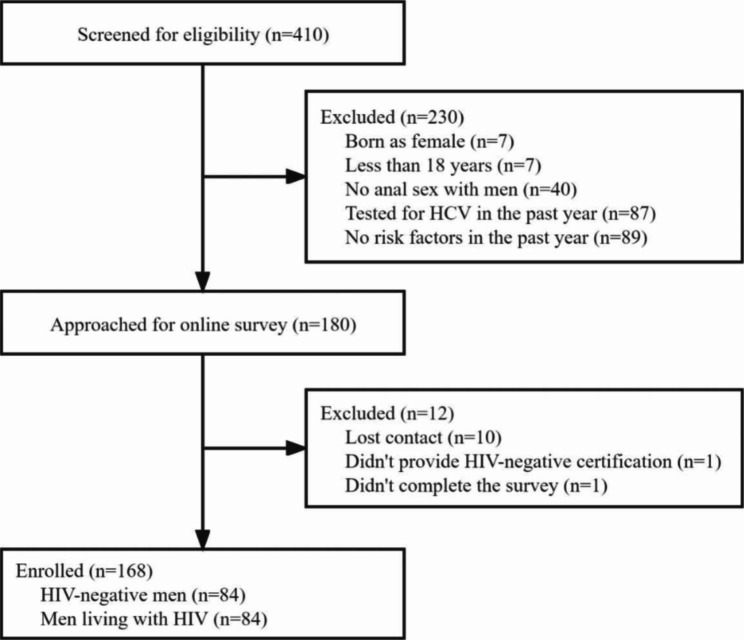
Flowchart diagram of study population

Overall, 410 people were approached for screening. Of whom, 1 did not complete the baseline survey and 230 did not meet eligibility requirements (7 were born biologically as a female, 7 were less than 18 years old, 40 reported no anal sex with other men, 87 was tested for HCV in the past year, 89 had no designated risk factors in the past year). A total of 179 individuals completed the online survey. Among them, 11 were further excluded, as they were unwilling to provide contact information (n = 10) and HIV-negative testing report (n = 1). In total, 168 participants (84 HIV-negative men and 84 men living with HIV) were included in this study. (Fig. 1)

### Socio-demographic characteristics

Most individuals reported never married (87.5%), employed (61.9%), had attained at least a junior college education (78.0%), had a moderate annual income (58.9% annual income between 5651 and 15,100 USD), and disclosed their sexual orientation to health provider, family or friends (69.6%). Key social-demographic were similar between individuals living with HIV and those who were HIV negative, except for the age and sexual orientation. (Table 1)

**Table 1:** Baseline social-demographic and behavioral characteristics of MSM in China

Variable	Total (N = 168)	HIV-negative MSM (N = 84)	MSM living with HIV (N = 84)	Chi-square	P-value
**Age**				7.056	**0.008**
≤30	115(68.5)	66(78.6)	49(58.3) ^**^		
>30	53(31.5)	18(21.4)	35(41.7)		
**Residence**				0.000	1.000
Urban	157(93.5)	78 (92.9)	79(94.0)		
Rural	11(6.5)	6 (7.1)	5 (6.0)		
**Educational Background**				0.688	0.709
High school or below	37(22.0)	17 (20.2)	20 (23.8)		
Junior college	66(39.3)	32 (38.1)	34 (40.5)		
Bachelor’s degree and above	65(38.7)	35 (41.7)	30 (35.7)		
**Marital status**				0.000	1.000
Ever married	21(12.5)	10 (11.9)	11 (13.1)		
Never married	147(87.5)	74 (88.1)	73 (86.9)		
**Employment status**				1.265	0.531
Unemployed	41(24.4)	20 (23.8)	21 (25.0)		
Employed	104(61.9)	50 (59.5)	54 (64.3)		
Other	23(13.7)	14 (16.7)	9 (10.7)		
**Annual income**				1.414	0.493
≤5650 USD	47(28.0)	22 (26.2)	25 (29.8)		
5651–15,100 USD	99(58.9)	53 (63.1)	46 (54.8)		
>15,100 USD	22(13.1)	9 (10.7)	13 (15.5)		
**Sexual orientation**				11.798	**0.003**
Gay	132(78.6)	61 (72.6)	71 (84.5) ^**^		
Bisexual	25(14.9)	12 (14.3)	13 (15.5)		
Other	11(6.5)	11 (13.1)	0 (0.00)		
**Disclosure sexual orientation to health provider family or friends**				1.014	0.314
Never	51(30.4)	29(34.5)	22(26.2)		
Ever	117(69.6)	55(65.5)	62(73.8)		
**Number of male sexual partners in the past 3 months**				0.394	0.531
0–1	69(41.1)	37(44.0)	32(38.1)		
Multiple	99(58.9)	47(56.0)	52(61.9)		
**Ever had condomless anal sex**				1.358	0.244
No	3(1.8)	0(0.0)	3(3.6)		
Yes	165(98.2)	84(100.0)	81(96.4)		
**Condomless anal intercourse in the past 6 months** ^**a**^				2.721	0.099
No	36(26.9)	13(19.7)	23(33.8)		
Yes	98(73.1)	53(80.3)	45(66.2)		
**Ever had unprotected anal fisting**				1.181	0.277
No	160(95.2)	78(92.9)	82(97.6)		
Yes	8(4.8)	6(7.1)	2(2.4)		
**Ever had group sex**				0.062	0.803
No	150(89.3)	76(90.5)	74(88.1)		
Yes	18(10.7)	8(9.5)	10(11.9)		
**Ever used substances before or during sex**				0.215	0.643
No	78(46.4)	41(48.8)	37(44.0)		
Yes	90(53.6)	43(51.2)	47(56.0)		
**Ever tested for syphilis**				2.574	0.109
No	61(36.3)	36(42.9)	25(29.8)		
Yes	107(63.7)	48(57.1)	59(70.2)		
**Ever tested for chlamydia**				0.177	0.674
No	141(83.9)	72(85.7)	69(82.1)		
Yes	27(16.1)	12(14.3)	15(17.9)		
**Ever tested for HBV**				0.024	0.877
No	92(54.8)	47(56.0)	45(53.6)		
Yes	76(45.2)	37(44.0)	39(46.4)		
**Ever tested for gonorrhea**				0.000	1.000
No	133(79.2)	66(78.6)	67(79.8)		
Yes	35(20.8)	18(21.4)	17(20.2)		
**Ever diagnosed with a STI**					
No	147(87.5)	82(97.6)	65(77.4) ^**^	13.932	**< 0.001**
Yes	21(12.5)	2(2.4)	19(22.6)		

**P < 0.01. Data are n/N (%) unless otherwise indicated. a. This analysis was restricted to participants who had condomless anal intercourse in the past 6 months

### Knowledge about HCV

Among HIV-negative men, the mean of correct answer rate for the 14 items on HCV knowledge was 53.9%. Only 39.3% (33/84) had a good level knowledge. (Table 2) The highest correct response (67.9%, 57/84) were obtained for two questions ‘The consumption of alcohol by people with HCV can damage the liver’ and ‘Using new, never used needles, syringes and other equipment reduces the risk of HCV infection’. The most frequently incorrect response (26.2%, 22/84) was for ‘The HCV vaccine can be used to prevent new infections with this virus’. (Table 3) Comparisons of knowledge level by socio-demographic and behavioral characteristics showed that only one variable was significantly different: employment status (p < 0.05). (Table 2)

**Table 2:** Participant characteristics by different levels of knowledge and factors correlated with knowledge among men who have sex with men in China

Characteristics	HIV-negative MSM (N = 84)					MSM living with HIV (N = 84)				
	Knowledge					Knowledge				
	Low	Moderate	High	cOR (95%CI)	aOR (95%CI) ^b^	Low	Moderate	High	cOR (95%CI)	aOR (95%CI) ^b^
	**N (%)**	**N (%)**	**N (%)**			**N (%)**	**N (%)**	**N (%)**		
**Total**	21(25.0)	30(35.7)	33(39.3)			21(25.0)	26(31.0)	37(44.0)		
**Age**										
≤30	16(24.2)	26(39.4)	24(36.4)	ref		16(32.7)	13(26.5)	20(40.8)	ref	
>30	5(27.8)	4(22.2)	9(50.0)	0.8(0.3,2.7)		5(14.33)	13(37.1)	17(48.6)	2.9(0.9,8.9)	
**Residence**										
Urban	19(24.4)	27(34.6)	32(41.0)	ref		20(25.3)	24(30.4)	35(44.4)	ref	
Rural	2(33.3)	3(50.0)	1(16.7)	0.6(0.1,3.8)		1(20.0)	2(40.0)	2(40.0)	1.4(0.1,12.9)	
**Educational Background**										
High school or below	2(11.8)	8(47.1)	7(41.2)	ref		7(35.0)	8(40.0)	5(25.0)	ref	
Junior college	12(37.5)	10(31.3)	10(31.3)	0.2(0.0,1.1)		11(32.4)	10(29.4)	13(38.2)	1.1(0.4,3.6)	
Bachelor’s degree and above	7(20.0)	12(34.3)	16(45.7)	0.5(0.1,2.9)		3(10.0)	8(26.7)	19(63.3)	4.8(1.1,21.8)*	
**Marital status**										
Ever married	2(20.0)	4(40.0)	4(40.0)	ref		3(27.3)	4(36.4)	4(36.4)	ref	
Never married	19(25.7)	26(35.1)	29(39.2)	0.7(0.1,3.7)		18(24.7)	22(30.1)	33(45.2)	1.1(0.3,4.8)	
**Employment status**										
Unemployed	5(25.0)	3(15.0)	12(60.0) ^**†**^	ref		5(23.8)	9(42.9)	7(33.3)	ref	
Employed	9(18.0)	23(46.0)	18(36.0)	1.5(0.4,5.3)		15(27.8)	14(25.9)	25(46.3)	0.8(0.3,2.6)	
Other	7(50.0)	4(28.6)	3(21.4)	0.3(0.1,1.4)		1(11.1)	3(33.3)	5(55.6)	2.5(0.2,25.2)	
**Annual income**										
≤5650 USD	7(31.8)	3(13.6)	12(54.5)	ref		8(32.0)	8(32.0)	9(36.0)	ref	
5651–15,100 USD	12(22.6)	23(43.4)	18(34.0)	1.6(0.5,4.8)		12(26.1)	13(28.3)	21(45.7)	0.9(0.3,2.4)	
>15100USD	2(22.2)	4(44.4)	3(33.3)	1.6(0.3,10.0)		1(7.7)	5(38.5)	7(53.8)	3.1(0.6,17.2)	
**Disclosure sexual orientation to health provider, family or friends**										
Never	6(20.7)	13(44.8)	10(34.5)	ref	ref	12(54.5)	6(27.3)	4(18.2) ^**‡**^	ref	ref
Ever	15(27.3)	17(30.9)	23(41.8)	0.7(0.2,2.0)	0.3(0.1,1.5)	9(14.5)	20(32.3)	33(53.2)	7.1(2.4,21.2)***	7.0(1.9,26.0)**
**Number of male sexual partners in the past 3 months**										
0–1	11(29.7)	10(27.0)	16(43.2)	ref	ref	4(12.5)	9(28.1)	19(59.4) ^**†**^	ref	
Multiple	10(21.3)	20(42.6)	17(36.2)	1.6(0.6,4.2)	1.3(0.4,4.4)	17(32.7)	17(32.7)	18(34.6)	0.3(0.1,1.0)*	0.2(0.1,1.0)*
**Ever had condomless anal sex**										
No	0(0.0)	0(0.0)	0(0.0)	-	-	0(0.0)	1(33.3)	2(66.7)	**-**	**-**
Yes	21(25.0)	30(35.7)	33(39.3)	-	-	21(25.9)	25(30.8)	35(43.2)	**-**	**-**
**Condomless anal intercourse in the past 6 months** ^**a**^										
No	5(38.5)	1(7.7)	7(53.8)	ref	ref	4(17.4)	9(39.1)	10(43.5)	ref	ref
Yes	11(20.8)	21(39.6)	21(39.6)	2.4(0.7,8.8)	2.5(0.6,11.4)	13(28.9)	11(24.4)	21(46.7)	0.5(0.1,1.8)	8.6(0.2,3.9)
**Ever had unprotected anal fisting**										
No	20(25.6)	26(33.3)	32(41.0)	ref	ref	20(24.4)	25(30.5)	37(45.1)	ref	ref
Yes	1(16.7)	4(66.7)	1(16.7)	1.7(0.2,15.7)	0.4(0.0,4.7)	1(50.0)	1(50.0)	0(0.0)	0.3(0.0,5.4)	0.4(0.0,10.1)
**Ever had group sex**										
No	18(23.7)	28(36.8)	30(39.5)	ref	ref	18(24.3)	22(29.7)	34(45.9)	ref	ref
Yes	3(37.5)	2(25.0)	3(37.5)	0.5(0.1,2.4)	0.1(0.0,1.0)	3(30.0)	4(40.0)	3(30.0)	0.8(0.2,3.2)	1.2(0.2,5.8)
**Ever used substances before or during sex**										
No	12(29.3)	11(26.8)	18(43.9)	ref	ref	12(32.4)	8(21.6)	17(45.9)	ref	ref
Yes	9(20.9)	19(44.2)	15(34.9)	1.6(0.6,4.2)	1.4(0.4,5.1)	9(19.1)	18(38.3)	20(42.6)	2.0(0.7,5.5)	3.7(1.1,13.1)*
**Ever tested for syphilis**										
No	10(27.8)	13(36.1)	13(36.1)	ref	ref	11(44.0)	7(28.0)	7(28.0) ^**†**^	ref	ref
Yes	11(22.9)	17(35.4)	20(41.7)	1.3(0.5,3.5)	1.1(0.3,4.0)	10(16.9)	19(32.2)	30(50.8)	3.9(1.4,10.9)*	3.1(0.8,12.0)
**Ever tested for chlamydia**										
No	18(25.0)	25(34.7)	29(40.3)	ref	ref	20(29.0)	19(27.5)	30(43.5)	ref	ref
Yes	3(25.0)	5(41.7)	4(33.3)	1.0(0.2,4.1)	1.1(0.2,6.0)	1(6.7)	7(46.7)	7(46.7)	5.7(0.7,46.4)	5.5(0.6,53.5)
**Ever tested for HBV**										
No	11(23.4)	18(38.3)	18(38.3)	ref	ref	18(40.0)	17(37.8)	10(22.2) ^**‡**^	ref	ref
Yes	10(27.0)	12(32.4)	15(40.5)	0.8(0.3,2.2)	0.7(0.2,2.6)	3(7.7)	9(23.1)	27(69.2)	8.0(2.1,30.0)**	7.3(1.6,32.7)**
**Ever tested for gonorrhea**										
No	18(27.3)	20(30.3)	28(42.4)	ref	ref	19(28.4)	19(28.4)	29(43.3)	ref	ref
Yes	3(16.7)	10(55.6)	5(27.8)	1.9((0.5,7.3)	1.9(0.4,9.6)	2(11.8)	7(41.2)	8(47.1)	3.0(0.6,14.2)	2.7(0.5,16.0)
**Ever diagnosed with a STI**										
No	20(24.4)	30(36.6)	32(39.0)	ref	ref	17(26.2)	19(29.2)	29(44.6)	ref	ref
Yes	1(50.0)	0(0.0)	1(50.0)	0.3(0.0,5.4)	0.1(0.0,2.9)	4(21.1)	7(36.8)	8(42.1)	1.3(0.4,4.6)	2.0(0.5,8.2)

^**†**^ Chi-square p-value < 0.05, ^**‡**^ Chi-square p-value < 0.001;

cOR: crude odds ratio; aOR: adjusted odds ratio; CI: confidence interval; *P < 0.05, **P < 0.01, ***P < 0.001

^**a**^ This analysis was restricted to participants who had condomless anal intercourse in the past 6 months

^**b**^ Multivariable logistic regression adjusted for age, region of residence, education, marital status, and income

**Table 3:** Knowledge related to HCV among MSM in China

Variable	HIV-negative MSM (N = 84)	MSM living with HIV (N = 84)	Chi-square	P-value
**Transmission routes**				
***People who inject drugs with “used needles” are infected with HCV***			0.256	0.612
Correct	57(67.9)	61(72.6)		
Incorrect	27(32.1)	23(27.4)		
***May be infected with HCV through sexual contact***			0.026	0.872
Correct	53(63.1)	55(65.5)		
Incorrect	31(36.9)	29(34.5)		
***The baby may be infected by a mother who was infected with HCV during delivery***			0.401	0.526
Correct	49(58.3)	54(64.3)		
Incorrect	35(41.7)	30(35.7)		
***HCV infection may occur during tattooing or piercing***			0.222	0.637
Correct	48(57.1)	52(61.9)		
Incorrect	36(42.9)	32(38.1)		
***People who use nasal cocaine are at risk of transmitting HCV through the use of shared straws, rolled up banknotes, etc.***			0.000	1.000
Correct	49(58.3)	49(58.3)		
Incorrect	35(41.7)	35(41.7)		
***People with HCV can safely share their toothbrushes and razors with others***			2.423	0.120
Correct	53(63.1)	42(50.0)		
Incorrect	31(36.9)	42(50.0)		
***People who underwent transfusion of blood products, may have been infected with HCV***			1.535	0.215
Correct	41(48.8)	50(59.5)		
Incorrect	43(51.2)	34(40.5)		
***HCV can be spread by sharing kitchenware (cups, plates, cutlery, etc.)***			0.894	0.345
Correct	37(44.0)	30(35.7)		
Incorrect	47(56.0)	54(64.3)		
**Prevention strategies on HCV**				
***Using new, never used needles, syringes and other equipment reduces the risk of HCV infection***			0.028	0.868
Correct	57(67.9)	59(70.2)		
Incorrect	27(32.1)	25(29.8)		
***The HCV vaccine can be used to prevent new infections with this virus***			5.696	**0.017**
Correct	22(26.2)	9(10.7) ^*^		
Incorrect	62(73.8)	75(89.3)		
**Treatment of HCV**				
***The consumption of alcohol by people with HCV can damage the liver***			2.363	0.124
Correct	55(65.5)	65(77.4)		
Incorrect	29(34.5)	19(22.6)		
***People infected with HCV may not be aware of the infection for many years***			0.648	0.421
Correct	51(60.7)	57(67.9)		
Incorrect	33(39.3)	27(32.1)		
***Effective HCV antiviral therapy can completely eliminate the virus from the patient’s blood***			4.721	**0.030**
Correct	30(35.7)	45(53.6) ^*^		
Incorrect	54(64.3)	39(46.4)		
***People after successful HCV antiviral therapy and viral eradication cannot be re-infected with this virus***			0.658	0.417
Correct	32(38.1)	26(31.0)		
Incorrect	52(61.9)	58(69.0)		
***The mean correct answer rates***	53.9	55.6		

*P < 0.05; Data are n/N (%) unless otherwise indicated

For men living with HIV, the mean rate was 55.6%. A total of 44.0% (37/84) had a good level of knowledge. (Table 2) The highest correct response (77.4%, 65/84) was obtained for the question ‘The consumption of alcohol by people with HCV can damage the liver’. The least correct response (10.7%, 9/84) was for ‘The HCV vaccine can be used to prevent new infections with this virus’. (Table 3) Comparisons of knowledge level by participants characteristics showed that four variables were significantly different in men living with HIV: the status of sexual orientation disclosure, number of sexual partners in the past 3 months, syphilis and HBV testing history (P < 0.05). (Table 2)

Comparing with MSM living with HIV, more HIV-negative men correctly reported that HCV cannot be prevented by vaccination (26.2% versus 10.7%, p < 0.05), but more incorrectly answered that HCV treatment did not eradicate the virus (35.7% versus 53.6%, p < 0.05). (Table 3)

### Attitude towards HCV

For HIV-negative men, nearly one-third (32.1%, 27/84) had a positive attitude towards HCV. (Table 4) Half (50.0%, 42/84) disagreed with the statement that ‘I would not want to be friends with someone with HCV’, which obtained the highest positive attitude score. More than a quarter (27.3%, 23/84) agreed with the statement that ‘I would feel uncomfortable having a conversation with someone who had HCV’, which obtained the highest negative attitude score (Table 5). Comparisons of attitudes level by participant characteristics showed that two variables were significantly different in HIV-negative men: substances use status and number of sexual partners in the past 3 months (p < 0.05). (Table 4)

**Table 4:** Participant characteristics by different levels of attitudes and factors correlated with attitudes among men who have sex with men in China

Characteristics	HIV-negative MSM (N = 84)					MSM living with HIV (N = 84)				
	Attitudes					Attitudes				
	Negative	Netural	Positive	cOR (95%CI)	aOR (95%CI) ^b^	Negative	Netural	Positive	cOR (95%CI)	aOR (95%CI) ^b^
	**N (%)**	**N (%)**	**N (%)**			**N (%)**	**N (%)**	**N (%)**		
**Total**	43(51.2)	14(16.7)	27(32.1)			29(34.5)	20(23.8)	35(41.7)		
**Age**										
≤30	31(47.0)	11(16.7)	24(36.4)	ref		16(32.7)	12(24.5)	21(42.9)	ref	
>30	12(66.7)	3(16.7)	3(16.7)	0.4(0.1,1.3)		13(37.1)	8(22.9)	14(40.0)	0.8(0.3,2.0)	
**Residence**										
Urban	38(48.7)	14(17.9)	26(33.3)	ref		28(35.4)	17(21.5)	34(43.0)	ref	
Rural	5(83.3)	0(0.0)	1(16.7)	0.2(0.0,1.7)		1(20.0)	3(60.0)	1(20.0)	2.2(0.2,20.6)	
**Educational Background**										
High school or below	11(64.7)	3(17.6)	3(17.6)	ref		9(45.0)	8(40.0)	3(15.0) ^**†**^	ref	
Junior college	18(56.3)	4(12.5)	10(31.3)	1.4(0.4,4.8)		12(35.3)	8(23.5)	14(41.2)	1.5(0.5,4.6)	
Bachelor’s degree and above	14(40.0)	7(20.0)	14(40.0)	2.8(0.8,9.2)		8(26.7)	4(13.3)	18(60.0)	2.25(0.7,7.4)	
**Marital status**										
Ever married	6(60.0)	3(30.0)	1(10.0)	ref		7(63.6)	2(18.2)	2(18.2)	ref	
Never married	37(50.0)	11(14.9)	26(35.1)	1.5(0.4,5.8)		22(30.1)	18(24.7)	33(45.2)	4.1(1.1,15.3)*	
**Employment status**										
Unemployed	13(65.0)	4(20.0)	3(15.0)	ref		9(42.9)	2(9.5)	10(47.6)	ref	
Employed	21(42.0)	8(16.0)	21(42.0)	2.6(0.9,7.5)		18(33.3)	15(27.8)	21(38.9)	1.5(0.5,4.2)	
Other	9(64.3)	2(14.3)	3(21.4)	1.0(0.2,4.3)		2(22.2)	3(33.3)	4(44.4)	2.6(0.4,15.8)	
**Annual income**										
≤5650 USD	13(59.1)	4(18.2)	5(22.7)	ref		9(36.0)	7(28.0)	9(36.0)	ref	
5651–15,100 USD	23(43.4)	9(17.0)	21(39.6)	1.9(0.7,5.2)		18(39.1)	9(19.6)	19(41.3)	0.9(0.3,2.4)	
>15100USD	7(77.8)	1(11.1)	1(11.1)	0.4(0.1,2.5)		2(15.4)	4(30.8)	7(53.8)	3.1(0.6,17.2)	
**Disclosure sexual orientation to health provider, family or friends**										
Never	12(41.4)	4(13.8)	13(44.8)	ref	ref	8(36.4)	4(18.2)	10(45.5)	ref	ref
Ever	31(56.4)	10(18.2)	14(25.5)	0.5(0.2,1.4)	0.4(0.1,1.4)	21(33.9)	16(25.8)	25(40.3)	1.1(0.4,3.1)	1.1(0.3,3.3)
**Number of male sexual partners in the past 3 months**										
0–1	27(73.0)	6(16.2)	4(10.8) ^**‡**^	ref	ref	13(40.6)	7(21.9)	12(37.5)	ref	ref
Multiple	16(34.0)	8(17.0)	23(48.9)	5.2(2.0,13.4)***	5.8(1.9,18.1)**	16(30.8)	13(25.0)	23(44.2)	1.5(0.6,3.9)	1.2(0.4,3.4)
**Ever had condomless anal sex**										
No	0(0.0)	0(0.0)	0(0.0)	**-**	**-**	0(0.0)	1(33.3)	2(66.7)	-	-
Yes	43(51.2)	14(16.7)	27(32.1)	**-**	**-**	29(35.8)	19(23.5)	33(40.7)	-	-
**Condomless anal intercourse in the past 6 months** ^**a**^										
No	7(53.8)	3(23.1)	3(23.1)	ref	ref	7(30.4)	6(26.1)	10(43.5)	ref	ref
Yes	20(37.7)	9(17.0)	24(45.3)	1.9(0.6,6.5)	2.0(0.5,8.0)	12(26.7)	12(26.7)	21(46.7)	1.2(0.4,3.6)	1.4(0.3,4.4)
**Ever had unprotected anal fisting**										
No	41(52.6)	14(18.0)	23(29.5)	ref	ref	27(32.9)	20(24.4)	35(42.7)	-	-
Yes	2(33.3)	0(0.0)	4(66.7)	2.2(0.4,12.8)	3.0(0.3,34.8)	2(100.0)	0(0.0)	0(0.0)	-	-
**Ever had group sex**										
No	39(51.3)	13(17.1)	24(31.6)	ref	ref	24(32.4)	18(24.3)	32(43.2)	ref	ref
Yes	3(37.5)	1(12.5)	3(37.5)	1.1(0.2,4.5)	0.7(0.1,3.9)	5(50.0)	2(20.0)	3(30.0)	0.5(0.1,1.8)	0.5(0.1,2.2)
**Ever used substances before or during sex**										
No	27(65.9)	6(14.6)	8(19.5) ^**†**^	ref	ref	18(48.6)	6(16.2)	13(35.1) ^**†**^	ref	ref
Yes	16(37.2)	8(18.6)	19(44.2)	3.3(1.3,8.0)*	3.1(1.0,9.4)*	11(23.4)	14(29.8)	22(46.8)	3.1(1.2,7.9)*	3.1(1.1,9.0)*
**Ever tested for syphilis**										
No	15(41.7)	7(19.4)	14(38.9)	ref	ref	9(36.0)	5(20.0)	11(44.0)	ref	ref
Yes	28(58.3)	7(14.6)	13(27.1)	0.5(0.2,1.2)	0.8(0.3,2.2)	20(33.9)	15(25.4)	24(40.7)	1.1(0.4,2.9)	1.3(0.4,4.4)
**Ever tested for chlamydia**										
No	36(50.0)	13(18.1)	23(31.9)	ref	ref	25(36.2)	16(23.2)	28(40.6)	ref	ref
Yes	7(58.3)	1(8.3)	4(33.3)	0.7(0.2,2.5)	0.9(0.2,3.8)	4(26.7)	4(26.7)	7(46.7)	1.6(0.4,5.4)	1.8(0.4,7.6)
**Ever tested for HBV**										
No	20(42.6)	8(17.0)	19(40.4)	ref	ref	17(37.8)	12(26.7)	16(35.6)	ref	ref
Yes	23(62.2)	6(16.2)	8(21.6)	0.5(0.2,1.1)	0.8(0.3,2.5)	12(30.8)	8(20.5)	19(48.7)	1.4(0.6,3.4)	1.6(0.5,4.5)
**Ever tested for gonorrhea**										
No	31(47.0)	13(19.7)	22(33.3)	ref	ref	23(34.3)	16(23.9)	28(41.8)	ref	ref
Yes	12(66.7)	1(5.6)	5(27.8)	0.4(0.1,1.3)	0.7(0.2,2.7)	6(35.3)	4(23.5)	7(41.2)	1.0(0.3,2.9)	1.0(0.3,3.6)
**Ever diagnosed with a STI**										
No	42(51.2)	13(15.9)	27(32.9)	ref	ref	23(35.4)	14(21.5)	28(43.1)	ref	ref
Yes	1(50.0)	1(50.0)	0(0.0)	1.1(0.1,17.4)	3.5(0.1,123.6)	6(31.6)	6(31.6)	7(36.8)	1.2(0.4,3.5)	1.4(0.4,4.6)

^**†**^ Chi-square p-value < 0.05, ^**‡**^ Chi-square p-value < 0.001;

cOR: crude odds ratio; aOR: adjusted odds ratio; CI: confidence interval; *P < 0.05, **P < 0.01, ***P < 0.001

^**a**^ This analysis was restricted to participants who had condomless anal intercourse in the past 6 months

^**b**^ Multivariable logistic regression adjusted for age, region of residence, education, marital status, and income

**Table 5:** Attitudes towards people living with HCV among MSM in China

Variable	HIV-negative MSM (N = 84)	MSM living with HIV (N = 84)
***I would feel pity for someone with HCV***		
Strongly disagree	0(0.00)	4(4.8) ^**^
Disagree	11(13.1)	5(6.0)
Neutral	36(42.9)	25(29.8)
Agree	24(28.6)	23(27.4)
Strongly agree	13(15.5)	27(32.1)
***I would not want my child to attend school where one of the students had HCV***		
Strongly disagree	16(19.0)	18(21.4)
Disagree	14(16.7)	18(21.4)
Neutral	39(46.4)	30(35.7)
Agree	11(13.1)	9(10.7)
Strongly agree	4(4.8)	9(10.7)
***I would not want to work in an office where one of the people there had HCV***		
Strongly disagree	18(21.4)	26(31.0)
Disagree	17(20.2)	22(26.2)
Neutral	39(46.4)	24(28.6)
Agree	6(7.1)	5(6.0)
Strongly agree	4(4.8)	7(8.3)
***I would not want to go to a small neighborhood grocery store where the owner had HCV***		
Strongly disagree	20(23.8)	26(31.0) ^*^
Disagree	15(17.9)	26(31.0)
Neutral	36(42.9)	18(21.4)
Agree	10(11.9)	9(10.7)
Strongly agree	3(3.6)	5(6.0)
***I would feel uncomfortable wearing a sweater once worn by a person with HCV***		
Strongly disagree	11(13.1)	16(19.0)
Disagree	15(17.9)	20(23.8)
Neutral	36(42.9)	28(33.3)
Agree	16(19.0)	13(15.5)
Strongly agree	6(7.1)	7(8.3)
***I would feel uncomfortable sharing a meal with someone who has HCV***		
Strongly disagree	13(15.5)	20(23.8)
Disagree	22(26.2)	21(25.0)
Neutral	38(45.2)	29(34.5)
Agree	8(9.5)	8(9.5)
Strongly agree	3(3.6)	6(7.1)
***I would not want to be friends with someone with HCV***		
Strongly disagree	14(16.7)	28(33.3)
Disagree	26(31.0)	27(32.1)
Neutral	36(42.9)	24(28.6)
Agree	5(6.0)	2(2.4)
Strongly agree	3(3.6)	3(3.6)
***I would not employ someone with HCV to work for me***		
Strongly disagree	17(20.2)	20(23.8)
Disagree	22(26.2)	33(39.3)
Neutral	33(39.3)	21(25.0)
Agree	7(8.3)	5(6.0)
Strongly agree	5(6.0)	5(6.0)
***I would feel uncomfortable having a conversation with someone who had HCV***		
Strongly disagree	16(19.0)	29(34.5) ^*^
Disagree	26(31.0)	27(32.1)
Neutral	37(44.0)	19(22.6)
Agree	4(4.8)	4(4.8)
Strongly agree	1(1.2)	5(6.0)
***I would not kiss someone with HCV***		
Strongly disagree	14(16.7)	13(15.5)
Disagree	9(10.7)	16(19.0)
Neutral	38(45.2)	26(31.0)
Agree	16(19.0)	17(20.2)
Strongly agree	7(8.3)	12(14.3)
***I would not date someone with HCV***		
Strongly disagree	11(13.1)	18(21.4)
Disagree	20(23.8)	25(29.8)
Neutral	40(47.6)	19(22.6)
Agree	8(9.5)	15(17.9)
Strongly agree	5(6.0)	7(8.3)
***I would not marry someone with HCV***		
Strongly disagree	10(11.9)	14(16.7)
Disagree	18(21.4)	20(23.8)
Neutral	37(44.0)	26(31.0)
Agree	14(16.7)	16(19.0)
Strongly agree	5(6.0)	8(9.5)
***I would avoid rooming with someone with HCV***		
Strongly disagree	14(16.7)	13(15.5)
Disagree	18(21.4)	23(27.4)
Neutral	36(42.9)	26(31.0)
Agree	10(11.9)	14(16.7)
Strongly agree	6(7.1)	8(9.5)

*P < 0.05, **P < 0.01; Data are n/N (%) unless otherwise indicated

Among men living with HIV, a total of 41.7% (35/84) of the men reported a positive attitude towards people with HCV. (Table 4) Nearly two-thirds (65.5%, 55/84) disagreed with the statement that ‘I would feel uncomfortable having a conversation with someone who had HCV’, which obtained the highest positive attitude score. In addition, 34.5% (29/84) agreed with the statement that ‘I would not kiss someone with HCV’, and this statement showed the highest negative attitude score. (Table 5) Comparisons of attitudes level by participants characteristics showed that only two variables were significantly different in men living with HIV: recreational substances use status and education levels (p < 0.05). (Table 4)

Compared with HIV-negative men, men living with HIV significantly agreed more that they would feel pity for someone with HCV (59.5% versus 44.1%, p < 0.01). Moreover, men living with HIV significantly more often disagreed with two statements: ‘I would not want to go to a small neighborhood grocery store where the owner had HCV’ (62.0% versus 41.7%, p < 0.05), and ‘I would feel uncomfortable having a conversation with someone who had HCV’ (66.6% versus 50.0%, p < 0.05). (Table 5)

### Sexual behaviors and STD testing history

Most participants reported having engaged in condomless anal intercourse in the past 6 months (73.1%, 98/134). Over half reported having two or more male anal sex partners in the past three months (58.9%, 99/168), having substance use before or during sex (53.6%, 90/168). In terms of other STD testing, about two-thirds (63.7%, 107/168) had tested for syphilis, roughly half (45.2%, 76/168) had ever tested for HBV, and a fifth tested for chlamydia (16.1%, 27/168) or gonorrhea (20.8%, 35/168). The sexual behavior and testing history characteristics of HIV-negative respondents were comparable to men living with HIV, except for the STI diagnosis experience. (Table 1)

### Factors correlated with knowledge about HCV

In the multivariable ordinal logistic regression analyses adjusted for age, region of residence, education background, marital status, employment status, and monthly income, there was no factor significantly associated with the level of HCV knowledge in HIV-negative men.

For men who living with HIV, the odds of moving from a poor level of knowledge to a moderate or good level of knowledge among men who disclosed their sexual orientation were 7 times (aOR: 7.0, 95%CI: 1.9–26.0) greater than those did not. Two other factors were also positively associated with a higher odds of having a good level of knowledge: substances use before or during sex (aOR: 3.7, 95%CI: 1.1–13.1), and HBV testing history (aOR: 7.3, 95% CI: 1.6–32.7). The other factor of men who had multiple sexual partners was negatively associated with a higher likelihood of having a good level of knowledge (aOR: 0.2, 95% CI: 0.1-1.0). (Table 2).

### Factors correlated with attitude towards HCV

For HIV-negative men, the odds of moving from negative attitude towards people living with HIV to neutral or positive attitude in men with multiple sexual partners were 5.8 times (aOR: 5.8, 95% CI: 1.9–18.1) greater than those with only one or no sexual partners. Another factor was also positively associated with a higher odds of having a positive attitudes: substances use before or during sex (aOR: 3.1, 95% CI: 1.0-9.4).

For men living with HIV, only one factor was positively associated with a higher odds of having a positive attitudes: substances use before or during sex (aOR: 3.1, 95% CI: 1.1-9.0). (Table 4)

## Discussion

MSM are at high risk of HCV acquisition and transmission, especially for those living with HIV. Knowing about the knowledge and attitudes related to HCV among MSM are critical for designing tailored interventions to prevent and eliminate HCV. Our study is one of very limited studies evaluating the overall knowledge and attitudes about HCV infection among MSM in China. Findings in this study indicated that a much greater effort is needed to improve the knowledge and attitudes about HCV infection among Chinese MSM.

We found that many Chinese MSM did not have a good level of knowledge about the HCV irrespective of HIV infection status. The mean correct answer rates of HCV knowledge of HIV-negative men (53.9%) and men living with HIV (55.6%) in our study were lower than previously reported among HCV patients (77%) and HCV/HIV co-infected patients (76%) in Canada [20]. The low rates of knowledge suggests a relative lack of tailored HCV education campaigns for MSM. Most participants in our study maintained accurate knowledge on some aspect of HCV transmission, but some false beliefs that HCV is transmissible by sharing food or kitchenware with infected individuals still existed. In addition, over half of individuals in both subpopulations still harbored some misconceptions about HCV vaccination and reinfection, which may diminish threat perceived and lead to less implementation of preventive measures. Studies have shown that low self-perceived risk may contribute to increased incidence of HCV in marginalized populations [21, 22]. Therefore, a greater emphasis should be placed on health promotion and risk communication with MSM, as well as ongoing, comprehensive HCV educational programs should be available to address the knowledge differences.

Our study showed that negative attitudes towards HCV have been common in Chinese MSM, especially in HIV-negative MSM. These negative attitudes may be related to misconceptions and fear of HCV. HIV-negative men were more likely to socially exclude HCV patients compared with MSM living with HIV, which is consistent with a previous study conducted in Australian MSM [23]. Given the differences in attitudes between the two subpopulations, effective interventions for MSM should be tailored according to HIV status. Men living with HIV were more likely to feel pity for and socially accept HCV patients, demonstrating the possibility of developing interventions based on a common perception of vulnerability to HCV within this subpopulation. For HIV-negative MSM, eliminating misconceptions and reducing negative attitudes towards HCV should be the main focus of the strategy, placing particular emphasis on beneficial information in educational materials could be helpful, such as the curability of HCV and the safety of daily social contact with HCV patients.

In the present study, most HIV-negative MSM and MSM living with HIV have reported risky sexual practices such as condomless anal sex, multiple sexual partners and the use of mucosally administered recreational drugs. Sexual behaviors leading to mucosal trauma is the predominant route of HCV acquisition among MSM, especially in HIV-infected individuals [24, 25]. Meanwhile, we found that over one-third of both HIV-negative MSM and MSM living with HIV did not know that HCV can be transmitted through sexual contact. This misunderstanding may hinder the use of effective HCV risk reduction strategies and increase propensities for high-risk sexual behaviors, which may explain the higher HCV burden in the MSM population. These findings highlight that HCV prevention efforts targeting MSM should also be focused on activating behavioral changes. However, current behavioral interventions that have been shown to be effective in lowering risky acts still suffer from low uptake, [26, 27] and may be insufficient as a stand-alone prevention strategy in reducing HCV transmission and infection [28]. Thus, multicomponent packages of evidence-based behavioral, educational and structural interventions (e.g., decreasing stigma and discrimination related to being gay) must be assembled to be appropriate, acceptable, and deliverable to the MSM population, so as to improve dissemination and eventual uptake of HCV interventions among this marginalized population [29, 30].

There are several limitations in this study. First, as all collected data were self-reported, social desirability bias may be present. However, we anticipate that this bias to be minimal as the survey was anonymous. Second, online survey might cause selection bias in the study, since recruited participants were primarily MSM who were young and well educated [31]. Nevertheless, our empirical generalizability research suggested that the results were similar when the online survey was quantitatively generalized to a national, cross-sectional survey dataset on MSM in China [32]. Third, this study was cross-sectional, therefore there was no causal relationships can be inferred. Fourth, our study recruited participant with a relatively small sample size, this may limit the statistical inference and generalizations of the results. However, according to a previous study that when sample size is 10 times greater than the number of variables, the power of the result was enough [33].

In conclusion, many Chinese MSM did not have a good level of knowledge and positive attitudes about HCV irrespective of HIV infection status. Tailored public health campaigns are required to ensure that MSM possess adequate and accurate knowledge. Given the negative attitudes can contribute to stigma and isolation, more research is required to inform how best to address the negative attitudes.

### Electronic supplementary material

Below is the link to the electronic supplementary material.

Supplementary Material 1

## Data Availability

The datasets used and/or analysed during the current study are available from the corresponding author on reasonable request.

## List of abbreviations

CBOs: Community-based organizations
DAA: Direct-acting antiviral
HBV: Hepatitis B virus
HCV: Hepatitis C virus
HIV: Human immunodeficiency virus
MSM: Men who have sex with men
RCT: Randomized controlled trials
STI: Sexually transmitted infection
SVR: Sustained virologic response
WHO: World Health Organization
